# Taxonomic review of the deep water flathead genus *Parabembras* with description of the new species *Parabembras multisquamata* from the western Pacific Ocean (Teleostei, Parabembridae)

**DOI:** 10.3897/zookeys.740.21729

**Published:** 2018-02-27

**Authors:** Yoshiaki Kai, Ronald Fricke

**Affiliations:** 1 Maizuru Fisheries Research Station, Field Science Education and Research Center, Kyoto University, Nagahama, Maizuru, Kyoto 625-0086, Japan; 2 Im Ramstal 76, 97922 Lauda-Königshofen, Germany

**Keywords:** New species, *Parabembras curta*, *Parabembras multisquamata*, *Parabembras robinsoni*, Papua New Guiana, Philippines, taxonomy, Vanuatu

## Abstract

Three valid species of *Parabembras* are recognized: *P. curta*, *P. robinsoni*, and the new species *P. multisquamata*. *Parabembras robinsoni* from the southwestern Indian Ocean (South Africa to Mozambique) is easily distinguishable from the other species in having eleven spines in the first dorsal fin, a distinct symphyseal knob on the lower jaw, two preocular spines, and a single lachrymal spine. *Parabembras multisquamata* from the southwestern Pacific (Vanuatu, Papua New Guinea) and the Philippines, and *P. curta*, known from the northwestern Pacific (southern Japan to South China Sea), are similar in sharing the absence of a symphyseal knob on the lower jaw, the presence of two lachrymal spines, and a single preocular spine, but the former is clearly distinguished from the latter in usually having 10 spines in the first dorsal fin (vs. eight or nine spines in *P. curta*), 9–11 supraocular spines (vs. 6–8 in *P. curta*), 40–44 pored lateral line scales (vs. 34–39 in *P. curta*), and the pectoral fin extending beyond the level of the anus (vs. not reaching to the level of the anus in *P. curta*).

## Introduction

The family Parabembridae, or deep water flatheads, comprises only two species, *Parabembras curta* (Temminck & Schlegel, 1843) from the northwestern Pacific Ocean, and *P. robinsoni* Regan, 1921, from the western Indian Ocean (Eschmeyer et al. 2017). The former species was originally placed in the genus *Bembras* Cuvier, 1829, but assigned by Bleeker (1874) to the then monotypic genus *Parabembras*. Jordan and Hubbs (1925), established the family as Parabembradidae [currently Parabembradidae (van der Laan et al. 2014)], but several authors synonymized Parabembridae with Bembridae (e.g., Knapp 1986; Nelson et al. 2016).

Imamura (1996; 2004) recognized Parabembridae as a valid family on the basis of a phylogenetic analysis based on osteological and myological characters. According to Imamura (1996), the family is diagnosed by the presence of spines and absence of tubercles on the dorsal surface of the head; the lower jaw projecting beyond the tip of the upper jaw; the separation of the ascending process and the remaining part of the premaxilla; the absence of free fin rays from the pectoral fin; three spines in the anal fin; about 35–40 scales in the lateral line; and the absence of a swim bladder.

We examined four relatively recently collected (1980–2012) specimens of a species of *Parabembras* from the waters of Vanuatu, Papua New Guinea, and the Philippines. These specimens differ from the two currently known species of the family by the spines on the head, number of dorsal-fin spines and pored lateral line scales, and several proportional measurements, and are described herein as a new species of *Parabembras*. In addition, the two congeneric species *P. curtus* and *P. robinsoni* are redescribed, and a key to the species of *Parabembras* is presented.

## Materials and methods

Counts and measurements follow Motomura (2004), except where otherwise noted. Body depths 1 and 2 are taken at the anterior insertions of the first and second dorsal fins, respectively; predorsal lengths 1 and 2 from tip the tip of the snout to the anterior insertion of the first and second dorsal fins, respectively; and the body width is measured between the uppermost part of the base of the right and left pectoral fins. Pored lateral line scales were counted from the first pored scale near the gill opening to the pored scale on the posterior margin of the hypural plate. The terminology of head spines follows Knapp et al. (2000). The standard length is abbreviated as SL. Characters given in the diagnosis of the genus were not repeated in the species descriptions. To provide an objectively defined score that summarizes the major components of variable measurements between specimens, a principal component analysis (PCA) was conducted on morphometric characters by using the function *prcomp* in the software package R 3.3.2. (R Core Team 2016). We removed the effect of changes in size by calculating the residuals from the linear regressions of changes in all variables on changes in SL (Revell et al. 2007). All variables were log-transformed prior to analysis.

The specimens examined in this study are deposited in the fish collections of the Natural History Museum, London (**BMNH**), Kyoto University, Kyoto and Maizuru (**FAKU**), Muséum national d’Histoire naturelle, Paris (**MNHN**), National Museum of Marine Biology and Aquarium, Taiwan (**NMMBA**), National Museum of Nature and Science, Tsukuba (**NSMT**), the National Taiwan University Museums, Taipei (**NTUM**), and the Museum of Comparative Zoology, Harvard University, Cambridge, Massachusetts (**MCZ**).

## Taxonomy

### Genus Parabembras

Taxon classificationAnimaliaScorpaeniformesParabembridae

Bleeker, 1874

Parabembras Bleeker, 1874: 370; Jordan and Richardson 1908: 644; Weber and de Beaufort 1911: 288; Jordan and Hubbs 1925: 281 (in family Parabembradidae); Barnard 1927: 936; Chu and Yin 1963: 478; Washington et al. 1984: 441; Knapp 1986: 481; Imamura 1996: 194 (in the monotypic family Parabembridae).

#### Type species.

*Bembras curtus* Temminck and Schlegel, 1843 by monotypy.

#### Diagnosis.

Body cylindrical, head somewhat depressed. Dorsal surface of head with spines only, tubercles absent. Ctenoid scales covering nape, postorbital, cheek, and opercular regions. Lower jaw projecting beyond upper jaw; dermal flap on posterior margin of maxillary. Two dorsal fins; first dorsal fin with 9 (rarely 8) to 11 spines; second dorsal fin with one spine and 8 or 9 soft rays. Anal fin with three robust spines and 5 soft rays. Pectoral fin without free rays. Pelvic fin with one spine and 5 soft rays, inserted below base of pectoral fin. Pored lateral line scales 34–44.

#### Remarks.

Imamura (1996) diagnosed the then monotypic family Parabembridae on the basis of dissection of a single species, *Parabembras curtus*. Because of the rarity of the other species, we could not confirm the status of internal diagnostic characters given by Imamura (1996). Although Nelson et al. (2016) placed *Parabembras* under family Bembridae with *Bembradium* Gilbert, 1905: the former is clearly distinguishable from the latter in having three anal-fin spines (vs. anal-fin spines absent). In the phylogenetic analysis, Imamura (2004) recovered a sister relationship between *Bembradium* and *Plectrogenium* Gilbert, 1905, forming the family Plectrogeniidae.

### Parabembras curta

Taxon classificationAnimaliaScorpaeniformesParabembridae

(Temminck & Schlegel, 1843)

Figs 1A , 2A

Bembras curtus Temminck & Schlegel, 1843: 42, pl. 16 (fig. 6–7), Nagasaki, Japan; Richardson 1846: 217, listed, Japan; Bleeker 1853: 11, listed, Japan; Bleeker 1855: 16, listed, Japan; Bleeker 1860: 49, listed, Japan; Günther 1860: 191, Sea of Japan; Boeseman 1947: 51, Nagasaki, Japan.
Parabembras curtus : Bleeker 1874: 370, new combination; Bleeker 1879: 12, listed, Japan; Jordan and Richardson 1908: 644, Kyushu, Japan (after Temminck and Schlegel, 1843); Weber and de Beaufort 1911: listed, 288; Jordan and Metz 1913: 54, listed, Busan, Korea; Jordan and Hubbs 1925: 281, Osaka, Japan; Reeves 1927: 12, listed, northeastern China and Korea; Liang 1951: 30, listed, Keelung, Taiwan; Kamohara 1952: 70, Tosa (= Kochi), Japan; Li 1955: 258, fig. 162, Yantai and Qingdao, Shandong, China; Nishibori 1959: 342, description of carotenoids; Chu and Jin 1963: 478, fig. 364, Zhejiang, East China Sea; Kamohara 1964: 77, listed, Tosa (=Kochi), Japan; Takegawa and Morino 1970: 386, listed, Wakasa Bay, Sea of Japan; Ochiai 1984: 321, pl. 288-A, in part, Pacific coast of Japan; Lindberg and Krasyukova 1987: 160, fig. 98 [after Temminck and Schlegel (1843)], Busan, Korea and East China Sea; Jean and Kuo 1988: 55, listed, northern Taiwan; Shen 1993: 260, pl. 66 (fig. 10), Taiwan; Suzuki and Kataoka 1997: 223, pl. 661, Owase, Mie, Japan; Randall and Lim 2000: 606, listed; Jin 2006: 490, fig. 233, Yellow Sea, Bohai Sea, northern Taiwan; Shao et al. 2008: 247, listed, Pintong, southern Taiwan; Shen and Wu 2011: 346, photo, Taiwan; Nakabo and Kai 2013: 718, 1950, key, Wakasa Bay and Kumano-nada, Japan southward to East China Sea; Shinohara et al. 2014: 247, listed, Sea of Japan; Yamamoto and Nagasawa 2015: 435, listed, East China and Yellow Seas.
Parabembras curta : Nakabo 2000: 614, Key, in part, Pacific coast of Japan; Shinohara et al., 2001: 318, listed, Tosa Bay; Nakabo 2002: 614 Key, in part, Pacific coast of Japan; Shinohara et al. 2005: 428, listed, Ryukyu Islands.
Bembradium roseum (not of Gilbert 1905): Shen and Wu 2011: 345, photo, Taiwan.

#### Material examined.

FAKU 12176, 12280, 12371, 14289, 41439, 41441, 41443, 41445–41447, 106.0–193.1 mm SL (10 specimens), East China Sea, coll. Matsui and Takai, 20 Oct. 1949; FAKU 34911, 145.5 mm SL (1), Yawatahama, Ehime, Japan, Kishida, Mar. 1962; FAKU 35093, 114.8 mm SL (1), Tosa Bay, Kochi, Japan,. K. Amaoka, Mar. 1962; FAKU 37892, 37893, 37897, 143.9–154.1 mm SL (3), Shimonoseki, Yamaguchi, Japan, N. Taniguchi, 10 Jun. 1965; FAKU 99918, 99919, 123.3–148.2 mm SL (2), Kii Ohshima, Wakayama, Japan; FAKU 101901, 123.3 mm SL (1), Tsushima, Nagasaki, Japan, 31 Jul. 1973; FAKU 144461, 144462, 94.8–135.4 mm SL, Dong-gang, Pingtung, Taiwan, F. Tashiro and M. Y. Lee; NMMBA 2820, 86.7 mm SL, Kaohsiung, Taiwan; NMMBA 8359, 87.9 mm SL, Dong-gang, Pingtung, Taiwan, 17 Mar. 2005; NMMBA 20313, 117.3 mm SL, Dong-gang, Pingtung, Taiwan, 25 May 2013.

**Figure 1.**
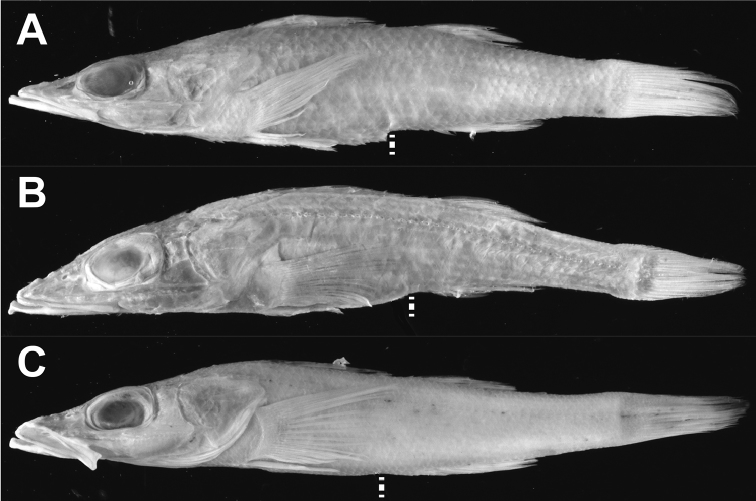
Lateral view of the three species of *Parabembras*; **A** *P. curta*, FAKU 41447, 143.5 mm SL **B** *P. robinsoni*, NSMT-P 129791, 165.1 mm SL **C** *P. multisquamata*, holotype, MNHN-IC-2008-1516, 167.3 mm SL. White line indicates anus.

**Figure 2.**
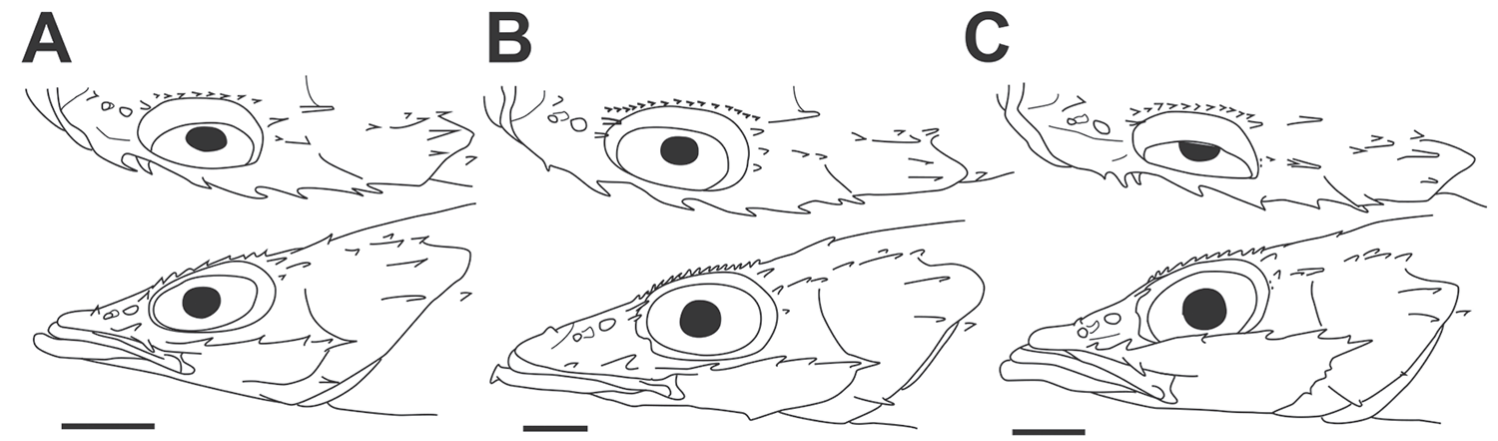
Lateral and dorsal views of the head of the three species of *Parabembras*; **A** *P. curta*, FAKU 41447, 143.5 mm SL **B** *P. robinsoni*, NSMT-P 129791, 165.1 mm SL **C** *P. multisquamata*, holotype, MNHN-IC-2008-1516, 167.3 mm SL. Bars equal to 10 mm.

#### Diagnosis.

*Parabembras curta* is distinguished from *P. robinsoni* in the absence of a symphyseal knob on the lower jaw (vs. distinct symphyseal knob in *P. robinsoni*), presence of a single preocular spine (vs. two preocular spines), and presence of two robust lachrymal spines (vs. a single robust lachrymal spine). It is further distinguished from *P. multisquamata*, in having 6–8 supraocular spines (vs. 9–11 supraocular spines in *P. multisquamata*); nine (rarely eight) spines in the first dorsal fin (vs. 10 spines); 34–39 pored lateral-line scales (vs. 40–44 pored lateral line scales).

#### Description.

Measurements are shown in Table 1. Body cylindrical, posteriorly weakly compressed, completely covered with ctenoid scales. Nasal spine simple, dorsoposteriorly directed. Anterior lachrymal with single robust spine, posterolaterally directed, in some specimens with small additional spine anteriorly; posterior lachrymal with sharp spine, posteroventrally directed. Single preocular spine present. Interorbital region narrow and weekly depressed, with 6–8 supraocular spines. Single postocular spine present, slightly larger than posteriormost supraocular spine. Suborbital ridge strongly or moderately developed, with three robust spines; tip of anteriormost spine below center of eye, second below posterior margin of eye, posteriormost below pterotic spine. Parietal spine robust and sharp, posteriorly directed. Three nuchal spines, one each on supratemporal, posttemporal, and supracleithrum, respectively; posttemporal in some specimens with additional spine. Pterotic with single sharp posteriorly directed spine. Preopercle with single robust posteriorly directed spine, in some specimens with supplementary spine; ventral margin of preopercle smooth. Opercle with upper and lower spines, directed slightly dorsoposteriorly and ventroposteriorly, respectively. Dermal flaps on head absent, except for flap on anterior nostril. Gill rakers blunt, usually four (in some specimens five) on upper limb; 11–13 (modally 12) on lower limb, including single (longest) raker at angle. Lateral line running parallel to dorsal contour of body, extending beyond caudal-fin base; 34–39 (modally 36) pored lateral line scales on body and 2–3 on caudal fin, each with single, robust tube.

**Table 1.** Counts and measurements of three species of *Parabembras*.

	*Parabembras curtus*		*P. robinsoni*		*P. multisquamus*			
	*n*=23		*n*=9		Holotype	Paratypes (*n*=3)		
	Range	Mean	Range	Mean		Range		Mean
Standard length (SL in mm)					167.3	146.4–186.9		
in % SL								
Head length	40.0–43.8	41.6	37.7–43.9	41.6	40.6	40.3–42.2		41.2
Snout length	9.7–11.0	10.4	10.4–12.4	11.2	10.6	10.1–10.7		10.4
Orbit diameter	11.3–14.2	12.5	11.7–14.1	12.6	12.0	12.3–13.9		13.0
Body depth 1	16.0–20.6	18.2	14.7–18.6	17.1	16.9	17.9–19.8		18.7
Body depth 2	15.2–18.1	16.6	13.6–16.2	14.8	16.1	16.5–17.4		17.0
Body width	15.9–19.6	17.8	13.9–18.8	16.2	15.1	14.6–16.8		15.8
Caudal peduncle depth	8.3–10.1	9.1	7.6–8.9	8.1	9.6	9.6–10.4		10.0
Upper jaw length	13.9–15.9	15.1	15.8–18.3	16.6	15.2	15.7–16.5		16.0
Predorsal fin length 1	37.8–41.2	39.3	37.4–41.3	39.2	40.3	38.1–40.9		39.6
Predorsal fin length 2	64.9–67.8	66.4	65.4–69.4	67.2	65.9	63.7–66.7		65.2
Preanal fin length	66.9–71.7	69.0	64.7–66.3	65.4	65.3	66.0–68.5		66.9
Preanal length	61.0–65.5	63.3	56.6–60.1	58.5	58.2	60.1–62.0		60.9
Prepelvic length	34.5–39.8	37.5	36.5–39.1	37.5	37.2	37.5–40.3		39.2
Pectoral fin length	22.5–28.4	26.3	22.6–27.3	25.4	26.4	24.2–28.5		26.6
Prepectoral fin length	36.9–40.9	38.4	38.8–42.2	40.0	38.9	38.9–39.6		39.3
Pelvic fin length	16.7–19.8	18.5	16.3–18.8	17.5	16.3	16.0–19.4		17.2
Pelvic fin spine length	9.5–14.0	11.5	9.6–11.5	10.5	10.6	10.2–13.0		11.5
Lengths of first dorsal fin								
1st dorsal-fin spine	2.4–3.9	2.9	1.7–4.4	2.7	2.6	2.1–3.0		2.6
2nd dorsal-fin spine	4.4–7.9	6.3	4.5–8.3	6.1	6.5	5.4–7.4		6.4
3rd dorsal-fin spine	10.0–14.0	11.8	8.3–12.3	10.2	11.1	10.6–11.3		11.0
4th dorsal-fin spine	14.4–20.6	16.5	11.6–15.3	14.0	14.6	14.7–14.7		14.7
5th dorsal-fin spine	15.0–20.9	17.7	12.5–15.8	14.1	14.8	13.7–15.1		14.4
Lengths of second dorsal fin								
1st dorsal-fin spine	13.3–17.3	15.2	10.6–14.1	12.2	11.1	10.4–12.5		11.3
1st dorsal-fin ray	15.4–18.1	16.6	13.1–15.9	15.0	15.7	14.9–16.7		16.0
1st anal-fin spine	4.0–7.1	5.6	3.4–5.1	4.1	5.1	5.2–5.7		5.5
2nd anal-fin spine	7.8–14.8	11.3	8.9–11.2	10.1	11.9	10.6–13.1		12.0
3rd anal-fin spine	8.1–11.1	9.3	7.9–10.1	9.2	10.1	9.1–11.2		10.1
1st anal-fin ray	12.1–16.9	14.2	12.2–14.2	13.3	13.9	13.3–15.5		14.3
Counts		Mode		Mode				Mode
Dorsal fin	VIII–IX-I, 8	IX-I, 8	X–XI-I, 8–9	XI-I, 9	X-I, 8	IX–X-I, 8		X-I, 8
Anal fin	III, 5	III, 5	III, 5	III, 5	III, 5	III, 5		III, 5
Pectoral fin	20–22	21	19–21	20	19	19–20		20
Pored lateral line scales	34–39	36, 37	38–41	40	44	40–44		43
Gill rakers	4–5+11–13	4+12	4–5+14–16	5+15	5+14	4–5+12		5+12

Mouth large, slightly oblique; maxilla reaching anterior rim of pupil; posterior margin of maxilla with distinct notch. Upper half of maxilla fitting within groove below suborbital ridge. Symphyseal knob absent from lower jaw. Upper and lower jaws with villiform teeth arranged in a band; vomer V-shaped, with villiform teeth; tooth band on palatine narrow.

First dorsal fin originating above level of pectoral-fin base, with usually nine spines (rarely eight), gradually increasing in length to fifth spine. First and second dorsal fins well separated. Second dorsal fin with one spine and eight soft rays; first soft ray somewhat longer than spine. Second dorsal and anal fins opposite each other, nearly equal in length and height; origin of latter slightly posterior to that of former. Caudal fin rounded. Pectoral fin rounded, upper half somewhat longer than lower half; its tip not reaching to level of anus, usually with 20–22 rays (modally 21), the lower 3–8 rays unbranched.

*Coloration*. In fresh condition, head and body reddish orange, white ventrally; faint dark red saddle below first and second dorsal fins, respectively; fins red, interradial membranes pale red; posterior half of caudal fin dark red. In preserved specimens, head and body pale brown; fins pale gray without any markings.

#### Geographical distribution.

**Figure 3.**
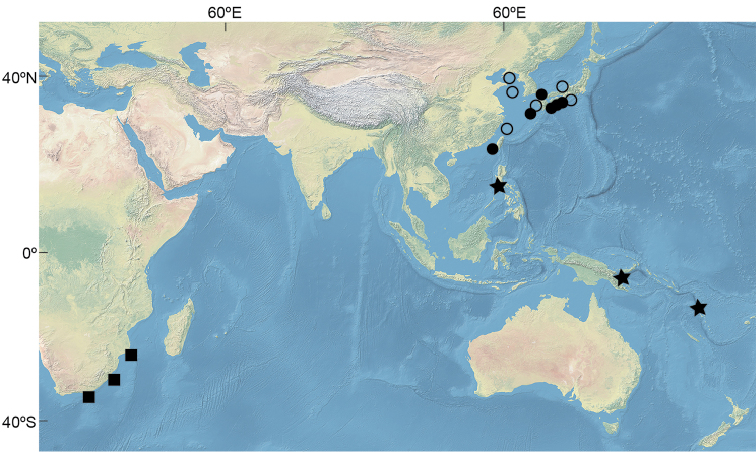
Distribution of the three species of *Parabembras* in the Indo-West Pacific. Circles, *P. curta*; squares, *P. robinsoni*; stars, *P. multisquamata*. Closed symbols - based on specimens examined in this study; open symbols - based on literature records. Map was made with layers from Natural Earth, free vector and raster map data at: http://naturalearthdata.com.

Known from the western Pacific Ocean; Wakasa Bay of Sea of Japan and Kumano-nada, Pacific coast of Japan and Korea south to China and Taiwan (East China Sea, Yellow Sea, Bohai, and northern part of South China Sea) (Fig. 3). Benthic, on sandy mud substrate from depths of 60–141 m (Chu and Jin, 1963; Nakabo and Kai, 2013; present study).

#### Remarks.

*Parabembras curta* was originally described as a member of *Bembras* by Temminck and Schlegel (1843) on the basis of the specimens collected in Japan 1823–1834 by Philipp von. Siebold and Heinrich Bürger. Boeseman (1947) reviewed the Siebold and Bürger’s collection, and designated RMNH-D 682 as the lectotype and RMNH-D 2057 as paralectotype of the species. According to Boeseman (1947), the lectotype has nine spines in the first dorsal fin and the paralectotype has eight spines. The figure of the species given in the original description is characterized as having eight spines in first dorsal fin, one spine and eight rays in the second dorsal fin, a single preocular spine, seven supraorbital spines, and two robust lacrimal spines. The photo of RMNH-D 682 published by Yamaguchi and Machida (2003) indicates clearly the absence of a symphyseal knob on the lower jaw.

The short description of *P. curta* given by Günther (1860) agrees with the present specimens in the nine spines in the first dorsal fin. Jordan and Hubbs (1925), who established the family Parabembridae (originally as Parabembradidae), described *P. curta* as having nine spines in the first dorsal fin and two spines and seven rays in the second dorsal fin. Although the count of spines in the second dorsal fin is not usual for any three species recognized here, the counts of pored lateral line scales (37), gill rakers on lower limb (11) and supraocular spines (6) agreed well with the present specimens of *P. curta*. The descriptions of *P. curta* from the East China Sea given by Li (1955), Chu and Jin (1963) and Jin (2006) and that from Kochi, Japan by Kamohara (1952) are referable to the species identified here with reference to the counts of dorsal-fin spine (9) and lateral line scales (35–40). Lindberg and Krasyukova (1987) recorded *P. curta* on the basis of four specimens from Busan (Korea) and the East China Sea. They characterized the species as having nine spines in the first dorsal fin and 36–38 lateral line scales, which agrees with counts in the present specimens of *P. curta*. Suzuki and Kataoka (1997) characterized *P. curta* from Mie Prefecture, Japan as having 10 spines in the first dorsal fin. Although the count of dorsal–fin spines was rather similar to *Parabembras multisquamata* described below, the pectoral fin of their specimen does not reach to anus, matching the condition of the present specimens of *P. curta*. In addition, the established distributional range of *P. curta* is close to the locality of Suzuki and Kataoka’s (1997) specimen. The short description of *P. curta* given by Ochiai (1984) apparently includes several species recognized here, because he assumed that *P. curta* was widely distributed in the Indo-West Pacific, and also presented a wide range in the count of dorsal-fin rays (IX–X-I, 7–9) (which apparently also includes *P. multisquamata* and *P. robinsoni*). The photograph provided by Ochiai (1984) agrees with the present specimens of *P. curta* in the short pectoral fin (not reaching to the anus) and absence of a symphyseal knob. The description of Shen (1993) apparently followed that of Ochiai (1984), but the photograph provided by him is referable to *P. curtus* recognized by the short pectoral fin (not reaching to the anus) and absence of a symphyseal knob. The keys and short descriptions of *P. curtus* presented by Nakabo (2000; 2002) also apparently followed Ochiai (1984). The images of *P. curta* and *Bembradium roseum* Gilbert, 1905 published by Shen and Wu (2011) are here both identified as *P. curta* in having nine spines in the first dorsal fin and three spines in the anal fin. However, their description of *B. roseum* does not agree with the specimen shown in their photograph which has 11 anal-fin rays.

The record of *P. curta* from the eastern Indian Ocean by Lin (1974: 26, western Indonesia) cannot be verified; specimens from these regions are needed to establish their identity. Although Krakstad et al. (2014: 74, 75) listed *P. curtus* from Myanmar, this record is not based on a species of *Parabembras*, but of *Bembras* (Peter Psomadakis, pers. comm.).

### Parabembras robinsoni

Taxon classificationAnimaliaScorpaeniformesParabembridae

Regan, 1921

Figs 1B , 2B

Parabembras robinsoni Regan, 1921: 418, KwaZulu-Natal, South Africa; Barnard 1927: 936; Smith 1949: 377, pl. 97, off Natal, South Africa; Smith 1961: 377, pl. 97, off Natal, South Africa; Smith 1965: 377, pl. 97, off Natal, South Africa; Knapp 1986: 482, pl. 29, fig. 154.1, Durban to southern Mozambique, South Africa; Schneider et al. 2005: 218, listed, Mozambique.
Parabembras curtus (not of Temminck and Schlegel 1843): Gilchrist 1922: 75, South Africa; Ochiai 1976: 105, pl. E. Afr-97, east coast of South Africa.
Parabembras sp.: Everett et al. 2015: 89, listed, Kenya, Tanzania, Mozambique.

#### Material examined.

BMNH 1921.3.1.19 (holotype of *P. robinsoni*, photo only), 24–35 km off Umvoti River, KwaZulu-Natal, South Africa [ca. 29°32'S, 31°36'E], depth 120–130 fathoms [219–238 m], R. Robinson, 1921; MCZ 130275, 127.0 mm SL (1), off Mozambique, 25°26'S, 34°19'E, 356 m depth, RS Algoa-014, 21 June 1994 (3D CTs only); NSMT-P 129786–129792, 126.7–176.1 mm SL (9 specimens), east coast of South Africa, 25°21'S, 34°20.5'E 326 m depth, 6 Dec. 1970.

#### Diagnosis.

*Parabembras robinsoni* is distinguished from its congeners in having usually 11 spines in the first dorsal fin (vs. 8–9 in *P. curta* and 9–10 in *P. multisquamata*), a distinct symphyseal knob in the lower jaw (vs. symphyseal knob absent in *P. curta* and *P. multisquamata*), two preocular spines (vs. single in *P. curta* and *P. multisquamata*), and single lachrymal spine (two in *P. curta* and *P. multisquamata*).

#### Description.

Measurements are shown in Table 1. Body cylindrical, posteriorly moderately compressed, completely covered with ctenoid scales. Anterior lachrymal with single robust spine, posterolaterally directed, in some specimens with small additional spine anteriorly; posterior lachrymal without spine. Two preocular spines present. Interorbital region narrow and slightly depressed, with more than 12 small spines, forming a serrated ridge. Single small postocular spine present. Suborbital ridge strongly or moderately developed, with three robust spines, tip of anteriormost spine below center of eye, second below posterior margin of eye; posteriormost below pterotic spine. Parietal spine sharp, posteriorly directed. Three nuchal spines, one each on supratemporal, posttemporal, and supracleithrum, respectively; posttemporal sometimes with additional spine. Pterotic with two sharp spines posteriorly directed. Posterior rim of orbit armed with small spines. Preopercle with single robust spine, posteriorly directed; usually with supplementary spine; ventral margin of preopercle smooth. Opercle with upper and lower spines, slightly directed dorsoposteriorly and posteriorly, respectively. Dermal flaps on head absent, except for flap on anterior nostril. Gill rakers blunt, usually 4–5 on upper limb; 14–16 (modally 15) on lower limb, including single (longest) raker at angle. Lateral line running parallel to dorsal contour of body, extending beyond caudal-fin base; 38–41 (modally 39) pored lateral-line scales on body and 2–3 on caudal fin, each with single, robust tube.

Mouth large, slightly oblique; maxilla reaching level of anterior rim of pupil; posterior margin of maxilla weakly notched. Upper half of maxilla fitting within groove below suborbital ridge. Lower jaw with distinct symphyseal knob. Upper and lower jaws with villiform teeth in a band; vomer V-shaped with villiform teeth; tooth band on palatine narrow.

First dorsal fin originating above level of pectoral-fin base, usually with eleven spines (in some specimens ten), gradually increasing in length to fifth spine. Last spine of first dorsal fin separated from penultimate spine without membrane, positioned midway between penultimate spine of first dorsal fin and insertion of second dorsal fin. Second dorsal fin with one spine and 8–9 soft rays; first soft ray slightly longer than spine. Second dorsal and anal fins directly opposite each other, nearly equal in length and height. Caudal fin rounded. Pectoral fin usually with 19–21 rays (modally 20), lower 4–7 rays unbranched; its rounded upper half somewhat longer than lower half, slightly extending beyond level of anus.

*Coloration*. In fresh condition, head and body reddish orange, white ventrally; fins red, interradial membranes pale red; distal margins of dorsal and anal fins dark red; posterior half of caudal fin dark red [based on pl. E. Afr-97 of Ochiai (1976)]. In preserved condition, head and body dark brown; fins pale gray; distal margin of second dorsal, anal and caudal fins dark brown.

#### Geographical distribution.

Western Indian Ocean, from Durban to at least southern Mozambique, along the east coast of South Africa at depths of 200–600 m (Knapp 1986). According to Everett et al. (2015), the species may be distributed along the East African coast north to Kenya; however, these records need confirmation.

#### Remarks.

*Parabembras robinsoni* was originally described by Regan (1921), characterized as having 10 spines in the first and one spine and nine soft-rays in the second dorsal fin. However, the last spine of the first dorsal fin, which is positioned between the penultimate ray of the first dorsal fin and the insertion of the second dorsal fin, is present in the holotype (BMNH 1921.3.1.19), which has eleven dorsal-fin spines, a distinct symphyseal knob in the lower jaw and a single lachrymal spine; hence, the present specimens are identified as *P. robinsoni*.

Barnard (1927) and Smith (1949, 1961, 1985) reported *P. robinsoni* from off the coast of KwaZulu-Natal, South Africa as having ten or eleven spines in the first dorsal fin. The short description by Knapp (1986) also characterized *P. robinsoni* as having ten or eleven spines in the first dorsal fin and a symphyseal knob in the lower jaw. These characters agree well with the present specimens of *P. robinsoni*. In contrast, Gilchrist (1922) described *P. curta* from South Africa and considered *P. robinsoni* as a junior synonym of *P. curta*. However, he described the species as having two preocular spines, agreeing with the present specimens of *P. robinsoni*, but not with *P. curta*. Similarly, Ochiai (1976) described *P. curta* from the east coast of South Africa. His photograph clearly shows *P. robinsoni*, judging by the presence of a distinct symphyseal knob in the lower jaw. Ochiai assumed that *P. curta* was widely distributed in the Indo-West Pacific, but apparently he confused *P. curta* and *P. robinsoni*. Probably due to this report, some subsequent authors mistakenly reported an occurrence of *P. curta* in the Indian Ocean (e.g., Nakabo 2000, 2002; Yamada et al. 2007).

### Parabembras multisquamata sp. n.

Taxon classificationAnimaliaScorpaeniformesParabembridae

http://zoobank.org/9342A016-DC81-4F49-A88E-FA17F64D42B3

Figs 1C , 2C

Parabembras curtus (not of Temminck and Schlegel 1843): Fricke 2015, 4, fig. 8, Morobe Province, Papua New Guinea.

#### Holotype.

MNHN-IC-2008-1516, 167.3 mm SL, 15°4'12"S, 166°57'0"E, Big Bay, Espiritu Santo, Vanuatu, 408–444 m depth, R/V Alis, expedition name: SANTO 2006, Station: AT106, 15 Oct. 2006.

#### Paratypes.

Four specimens. MNHN-IC-1984-0687, 170.1 mm SL, 13°49'1.2"N, 120°51'0"E, off southwestern Luzon, Philippines, 299–320 m depth, expedition name: MUSORSTOM 2; Station: 26cp4, 23 Nov. 1980; MNHN-IC-2008-2443, 2009-0115, 158.3–186.9 mm SL, 15°4'21"S, 166°51'46.8"E, Big Bay, Espiritu Santo, Vanuatu, 350–400 m depth, R/V Alis, expedition name: BOA1; Station: CP2416, 6 Sep. 2005; NTUM 10690, 146.4 mm SL, Papua New Guinea, Morobe Province, 28 km east of Lae, 06°45'03.90"S, 147°14'40.44"E – 06°45'18.24"S, 147°14'03.26"E, 360 m depth, R/V Alis, expedition name: PAPUA NIUGINI; Station: CP3999, 10 Dec. 2012.

#### Diagnosis.

*Parabembras multisquamata* is distinguished from *P. robinsoni* in having two lachrymal spines and no symphyseal knob on the lower jaw. It is most similar to *P. curta*, but is clearly distinguished from the latter in having usually 10 first dorsal-fin spines (vs. 8 or 9 spines in *P. curta*), 9–11 supraocular spines (vs. 6–8 in *P. curta*), and 40–44 pored lateral line scales (vs. 34–39 in *P. curta*).

#### Description.

Measurements are shown in Table 1. Data of the holotype are given first, followed by data of the paratypes, in parentheses, if different from holotype. Body cylindrical, posteriorly weakly compressed, completely covered with ctenoid scales. Nasal spine simple, dorsoposteriorly directed. Anterior lachrymal with single robust spine, posterolaterally directed, with small additional spine anteriorly (without an additional spine in MNHN-IC-1984-0687); posterior lachrymal with sharp spine, directing posteroventrally. Single preocular spine present. Interorbital region narrow and weekly depressed, armed with 11 (9–11) supraocular spines. Single postocular spine present, somewhat larger than posteriormost supraocular spine. Suborbital ridge strongly developed (moderately developed), with three (three or four) robust spines; tip of anteriormost spine below center of eye, second below posterior margin of eye, third below pterotic spine (posteriormost on margin of preopercle). Three nuchal spines; one each on supratemporal, posttemporal, and supracleithrum, respectively. Pterotic with two sharp spines posteriorly directed. Posterior rim of orbit armed with small spines. Preopercle with single robust and several small supplementary spines, posteriorly directed; ventral margin of preopercle with three (0–5) tiny spines. Opercle with upper and lower spines, slightly dorsoposteriorly and ventoposteriorly directed, respectively. Dermal flaps on head absent, except for flap on anterior nostril. Gill rakers blunt, usually 5 (4–5) on upper limb; 14 (12–13) on lower limb, including single (longest) raker at angle. Lateral line running parallel to dorsal contour of body, extending beyond caudal-fin base; 44 (40–44) pored lateral-line scales on body and 2 (2–3) on caudal fin, each with single, robust tube.

Mouth large, slightly oblique; maxilla reaching anterior rim of pupil; posterior margin of maxilla with distinct notch. Upper half of maxilla fitting within groove below suborbital ridge. Symphyseal knob absent from lower jaw. Upper and lower jaws with villiform teeth arranged in a band; vomer V-shaped, with villiform teeth; tooth band on palatine narrow.

First dorsal fin originating above level of pectoral-fin base, usually with 10 spines (nine in MNHN-IC-2009-0115), gradually increasing in length to fifth (forth or fifth) spine. First and second dorsal fins well separated. Second dorsal fin with one spine and 8 soft rays; first soft ray somewhat longer than spine. Second dorsal and anal fins opposite each other, nearly equal in length and height; anterior insertion of latter slightly posterior to that of former. Caudal fin rounded. Pectoral fin rounded, upper half somewhat longer than lower half; its tip extending beyond level of anus, with 19 rays (19–21) of which lower 7 (6–7) rays unbranched.

*Coloration*. In fresh specimens [based on fig. 8 in Fricke (2015), NTUM 10690], head and body red, white ventrally; first dorsal fin dark red margined with black; second dorsal fin red with black marking; posterior half of caudal fin dark red; pectoral fin bright red; pelvic fin pale red. In preserved condition, head and body pale brown; first dorsal fin margined with black; second dorsal fin with dark brown marking.

#### Geographical distribution.

Known from the western Pacific Ocean, off southwestern Luzon, Philippines, Morobe Province of Papua New Guinea, and Espiritu Santo, Vanuatu. The new species was collected at depths of 299–444 m (Fig. 3).

#### Etymology.

The name *multisquamata* is derived from Latin *multus* meaning many and *squamatus* meaning scaled, in reference to the high number of pored lateral line scales. The name is an adjective, its ending following the feminine gender of the generic name *Parabembras*.

#### Remarks.

Fricke (2015) reported *P. multisquamata* as *P. curtus* (non Temminck and Schlegel 1843) from Morobe Province of Papua New Guinea on the basis of a single specimen, NTUM 10690, which is now one of the paratypes of *P. multisquamata*. Judging from the collection data, the record of *P. curta* from off southwestern Luzon, Philippines by Fourmanoir (1985: 46, as *P. curtus*) was based on MNHN-IC-1984-0687, one of the paratypes of *P. multisquamata*.

### Key to species of *Parabembras*

**Table:** 

1	Lower jaw with a distinct symphyseal knob; lachrymal with single robust spine [western Indian Ocean]	***Parabembras robinsoni***
–	Lower jaw without a symphyseal knob; lachrymal with 2 robust spines	**2**
2	First dorsal fin usually with 10 (rarely 9) spines, head with 9–11 supraocular spines, pored lateral line scales 40–44, pectoral fin extending beyond the level of anus [southwestern Pacific Ocean and the Philippines]	***Parabembras multisquamata***
–	First dorsal fin with 8 or 9 spines, head with 6–8 supraocular spines, pored lateral line scales 34–39, pectoral fin not reaching to the level of anus [northwestern Pacific Ocean]	***Parabembras curta***

## Discussion

**Figure 4.**
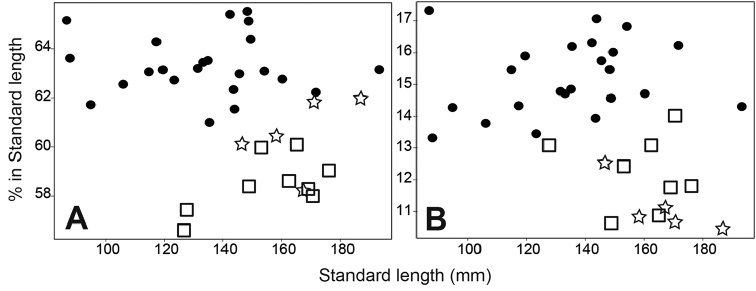
Comparison of selected morphometric characters of species of *Parabembras*; **A** proportion of preanal length **B** first spine of second dorsal fin. Circles, *P. curta*; squares, *P. robinsoni*; stars, *P. multisquamata*.

**Figure 5.**
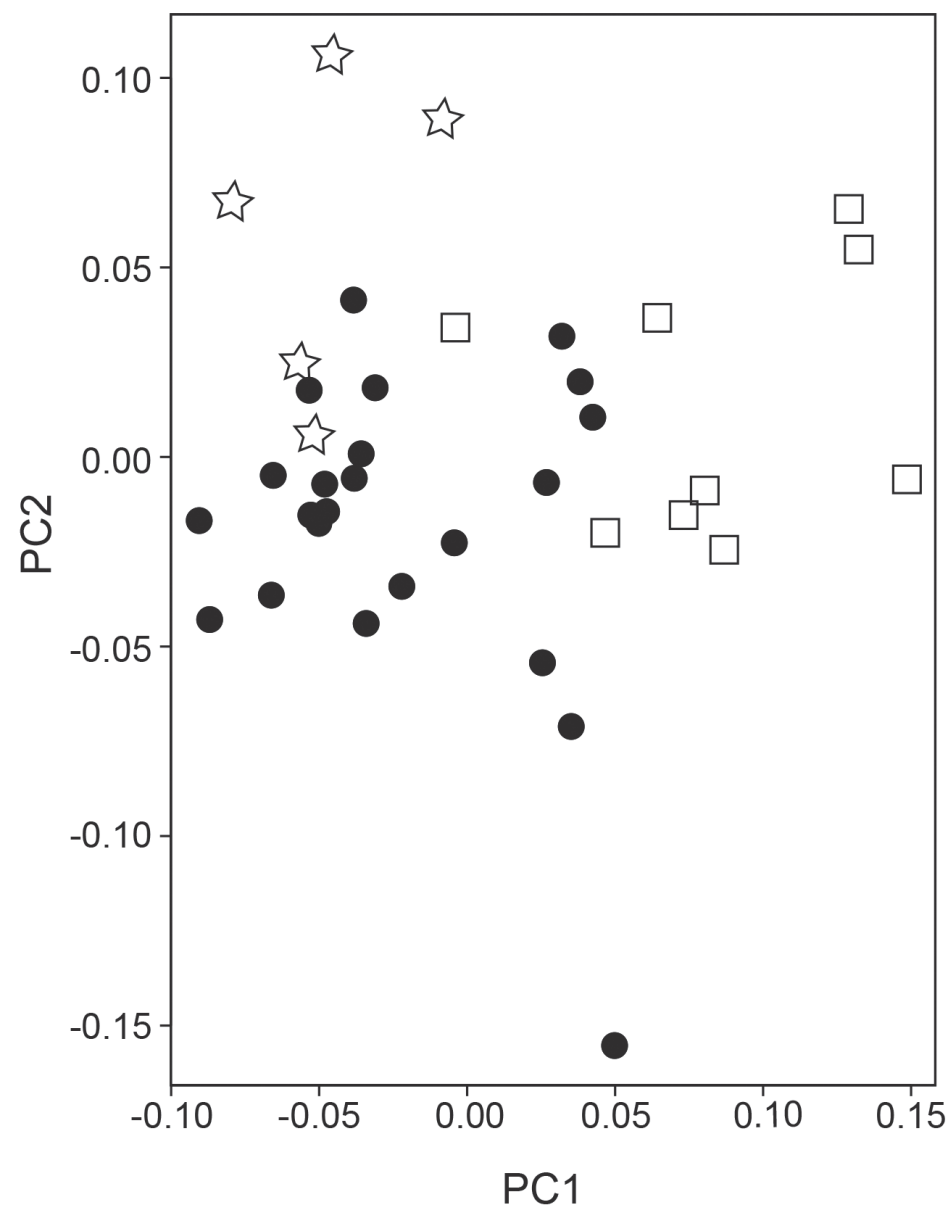
Plots of the first two principal components scores based on 20 body measurements of the three species of *Parabembras*. Circles, *P. curta*; squares, *P. robinsoni*; stars, *P. multisquamata*.

*Parabembras multisquamata* is most similar to *P. curta* in having two lachrymal spines and no symphyseal knob on the lower jaw, but is clearly distinguished as described above. These two species differ further in some morphometric characters, including the preanal length and the first spine of the second dorsal fin (Fig. 4). Furthermore, the pectoral fin of *P. multisquamata* extends beyond the level of the anus (vs. not reaching to the level of anus in *P. curta*). The PCA using 20 measurements (eight were eliminated due to the lack of data in some of the specimens) resulted in the rough separation of three species. The first and second principal components accounted for 34.4 % and 18.3 % of the variation. PC1 was heavily loaded on caudal peduncle depth, body depth 2, and length of second anal-fin spine, providing separation between *P. robinsoni* and the other two species (Fig. 5). PC2 was heavily loaded on length of second anal-fin spine, body width, and body depth 1, providing separation between *P. multisquamata* and the other two species. These results also support the existence of three species in *Parabembras*. The distributional ranges of the three species are not overlapped, suggesting that they speciated allopatrically.

## Supplementary Material

XML Treatment for Parabembras

XML Treatment for Parabembras curta

XML Treatment for Parabembras robinsoni

XML Treatment for Parabembras multisquamata
